# Wealth stratified inequalities in service utilisation of breast cancer screening across the geographical regions: a pooled decomposition analysis

**DOI:** 10.1186/s13690-020-00410-5

**Published:** 2020-06-10

**Authors:** Rashidul Alam Mahumud, Khorshed Alam, Syed Afroz Keramat, Andre M. N. Renzaho, Md. Golam Hossain, Rezwanul Haque, Gail M. Ormsby, Jeff Dunn, Jeff Gow

**Affiliations:** 1 School of Social Sciences and Psychology, Western Sydney University, Locked Bag 1797, Penrith, NSW 2751 Australia; 2 Translational Health Research Institute (THRI), Western Sydney University, Sydney, NSW Australia; 3 Health Economics and Policy Research, Centre for Health, Informatics and Economic Research, University of Southern Queensland, Toowoomba, QLD 4350 Australia; 4 School of Commerce, University of Southern Queensland, Toowoomba, QLD 4350 Australia; 5 Health Economics and Financing Research, International Centre for Diarrhoeal Disease Research, Dhaka, 1212 Bangladesh; 6 Department of Economics, American International University-Bangladesh (AIUB), Dhaka, 1212 Bangladesh; 7 Health and Epidemiological Research Group, Department of Statistics, University of Rajshahi, Rajshahi, 6205 Bangladesh; 8 Professional Studies, Faculty of Business, Education, Law and Arts, University of southern Queensland, Toowoomba, QLD 4350 Australia; 9 Cancer Research Centre, Cancer Council Queensland, Fortitude Valley, QLD 4006 Australia; 10 Prostate Cancer Research Foundation of Australia, St Leonards, NSW 2065 Australia; 11 School of Accounting, Economics and Finance, University of KwaZulu-Natal, Durban, 4000 South Africa

**Keywords:** Breast cancer screening, Decomposition analysis, Low-resource countries, Inequality

## Abstract

**Background:**

Breast cancer is the most commonly occurring cancer among women in low-resourced countries. Reduction of its impacts is achievable with regular screening and early detection. The main aim of the study was to examine the role of wealth stratified inequality in the utilisation breast cancer screening (BCS) services and identified potential factors contribute to the observed inequalities.

**Methods:**

A population-based cross-sectional multi-country analysis was used to study the utilisation of BCS services. Regression-based decomposition analyses were applied to examine the magnitude of the impact of inequalities on the utilisation of BCS services and to identify potential factors contributing to these outcomes. Observations from 140,974 women aged greater than or equal to 40 years were used in the analysis from 14 low-resource countries from the latest available national-level Demographic and Health Surveys (2008–09 to 2016).

**Results:**

The population-weighted mean utilisation of BCS services was low at 15.41% (95% CI: 15.22, 15.60), varying from 80.82% in European countries to 25.26% in South American countries, 16.95% in North American countries, 15.06% in Asia and 13.84% in African countries. Women with higher socioeconomic status (SES) had higher utilisation of BCS services (15%) than those with lower SES (9%). A high degree of inequality in accessing and the use of BCS services existed in all study countries across geographical areas. Older women, access to limited mass media communication, being insured, rurality and low wealth score were found to be significantly associated with lower utilisation of BCS services. Together they explained approximately 60% in the total inequality in utilisation of BCS services.

**Conclusions:**

The level of wealth relates to the inequality in accessing BCS amongst reproductive women in these 14 low-resource countries. The findings may assist policymakers to develop risk-pooling financial mechanisms and design strategies to increase community awareness of BCS services. These strategies may contribute to reducing inequalities associated with achieving higher rates of the utilisation of BCS services.

## Background

In recent years, there has been an alarming increase in the incidence of breast cancer in low- resource countries [1]. Although many cancer rates continue to be higher in more affluent countries, the incidence of breast cancer and breast cancer-related mortality rates are growing worldwide [2]. In low-resource countries over one million new cases of breast cancer are diagnosed yearly, and more than 70% of women die as a result of breast cancer and a lack of BCS services, mainly early detection through regular screening [3]. It is estimated that in 2020, approximately 1.7 million women will be diagnosed with breast cancer in low-resource countries [4]. The overall burden of breast cancer has been increasing amongst the most vulnerable populations in these countries. Globally, survival rates of breast cancer differ significantly [2]. The five-year survival rates for breast cancer are poorly documented for low-resource countries, but based on available figures, they are below 40% compared with developed countries at 82% [5, 6].

Early detection of breast cancer improves survival rates [7]. Breast cancer is preventable through early detection using established screening protocols [6, 8]. Timely and regular screening and early treatment have significantly reduced breast cancer mortality rates by 20–30% in adult women (over 45 years of age) in developed countries [8]. However, the use of BCS services is extremely low in developing countries [9]. These countries currently face the challenge of detecting and treating breast cancer effectively and appropriately within overstretched health budgets which do not prioritise women’s health service provision. Data on the incidence of breast-cancer is patchy in many low-resource countries.

Several factors determine the access to and the use of BCS services. For example, educational background, health insurance coverage, employment, and household income are significant [10, 11]. Other socioeconomic status (SES) related factors include social class, economic characteristics and position, and educational background [12], and the availability of health care facilities have also played a significant role in ensuring higher rates of BCS services usage [7, 11, 13]. Factors such as access to health care facilities, location (urban is better than rural), medical staff attitudes, cultural beliefs and religious affiliation have also influenced women’s utilisation of BCS [14–18]. Studies examining the influence of SES factors on the use of BCS services have been conducted in a range of settings [13, 19–23]. These studies all confirm that disadvantaged women are less likely to access and utilise available services compared to women from higher SES cohorts. Most of these studies have been descriptive in nature, lacking analytical rigour and usually covering only a small number of study participants and with limited study areas. Most of the studies have been conducted in developed nations. However, little attention has been paid to examining the role of inequality in determining which factors are significant in the use of BCS services. Therefore, the main aim of the study was to examine the role of wealth stratified inequality in the utilisation of BCS services. Using available data, this study included a sample of 140,974 women drawn from 14 developing countries across varied geographical areas to obtain a better understanding of these relationships in this study. Additionally, a regression-based decomposition approach was used to delineate these potential factors influencing the degree of inequality.

## Methods and materials

### Study design and data

The study was population-based and multi-country using a cross-sectional design using available standard Demographic and Health Survey (DHS) data. The DHSs are nationally-representative household surveys that provide data for a wide range of monitoring and impact evaluation indicators in the areas of population, health, and nutrition. Approval was received from DHS to use data. Data were extracted from the most recent DHS, covering 14 low-resource countries for the period from 2008 to 2016 [24–37]. The DHS surveys have been part of a long-standing worldwide program that includes individual and household-level, socio-demographic, health indicators and health care data in the context of low-resource countries. These national-level surveys, generally conducted every 3 years, capture information related to maternal and child health, mortality, fertility, family planning, and nutrition-related parameters.

A two-stage stratified cluster sampling was used. In the first stage, samples were selected from the main DHS sampling frame developed from enumeration areas. In the second stage, systematic random sampling was employed. The detailed information regarding survey sampling, quality control, management, and survey instruments are reported elsewhere [24–37]. Trained interviewers collected data using face-to-face interviews. Written consent was collected from the respondents before conducting the survey. The survey response rate varied between 85 and 95%. A sample was drawn from the DHS database for analysis, which resulted in a total of sample 140,974 women living in 14 low- resource countries. India had the highest proportion of participants (43,502 women, 31% of the total sample), followed by Egypt (18,254 women, 13% of the sample). The average (standard deviation) age of the participants was 49.54 (± 2.32) years.

### Study settings

Of the 90 countries where the DHS surveys have been implemented, BCS related questions in 18 countries (20.0%) [24, 38]. The common themes identified were disease knowledge, screening knowledge, screening practice, and screening outcomes. In this study, data on the utilisation of BCS services was used from the 14 low-resource countries during the period of 2008–2016 namely: Albania (2008–09), Burkina Faso (2010), Colombia (2015), Cote d’Ivoire (2011–12), Dominican Republic (2013), Egypt (2015), Honduras (2011–12), India (2015–16), Jordan (2012), Kenya (2015), Lesotho (2014), Namibia (2013), Philippines (2013), and Tajikistan (2012) [24–37]. However, Equatorial Guinea and Peru were excluded from the analysis because their data were not publicly accessible. Armenia was excluded because it lacked sufficient information related to the study variables. Brazil was excluded due to obsolete data in 1986. Countries were grouped across geographical regions according to the continent such as Africa (i.e., Kenya, Burkina Faso, Egypt, Lesotho, Namibia, Cote d’Ivoire), Asia (e.g., India, Philippines, Jordan, Tajikistan), Europe (e.g., Albania), North America (e.g., Honduras, Dominican Republic) and South America (e.g., Colombia).

### Participant’s inclusion criteria

The study participants were restricted to women aged 40 years or more at risk of developing breast cancer [39–44]. Several types of studies also used a similar inclusion criterion for epidemiological, observations and clinical studies [39–43]. This is because breast cancer diagnosis among younger women is more complex because their breast tissue is usually more dense compared to their older counterpart [40–42].

### Definition of study variables

#### Outcome variable

Participants were asked questions related to their utilisation of BCS services [38]. For example, or ‘have you ever had a mammogram?’ or ‘have you had a clinical breast cancer examination?’ Participants self-reported as their responses in the form of a dichotomous (‘yes’ or ‘no’) and this information was used as the outcome variable in the analytical exploration.

#### Explanatory variables

Explanatory variables were selected based on different criteria, including epidemiology and published studies on the utilisation of BCS and these data were examined for potential confounders [3, 7, 38, 44]. Explanatory variables were selected based on the available in the DHS data sets. The participants’ characteristics, including age, education, sex of household head and age at the time of respondent’s first birth, were selected as potential predisposing factors in the analyses. Age was grouped as follows: 40–44 years or ≥ 45 years. Participant’s educational background was categorised as: no education, primary education, secondary education or higher education. The head of the participant’s household was defined as ‘male’ if the participants lived in the male-dominated household, or ‘female’ if otherwise. The number of live births was classified as < 4 births, 4–5 births, or > 5 births. Participant’s mass media exposure was assessed by means of access to radio and television in the household. Health insurance coverage, body mass index, and wealth status were considered enabling factors. Health insurance coverage in households was dichotomous (‘yes’ if insured of the participants household or ‘no’ if uninsured). The height and body weight of the participants were measured by trained field research staff. Weight was measured once, with light clothing on and without shoes, by digital weighing scales placed on a flat surface. Height was measured once using a standard clinical height measuring scale with the participant standing without shoes. Body mass index (BMI) was calculated as the ratio of weight in kilograms (kg) to height in meters (m) squared (kg/m^2^). SES was based on the ownership of durable assets [45]. This method has been used in previous studies using DHS data from developing countries [38, 46, 47]. Each household’s characteristics (assets) were dichotomised (‘yes’ if present and ‘no’ if not). Country-specific principal components analysis (PCA) was performed using this ownership of durable assets [37]. Weights were estimated by factor scores derived from the first principal component in the PCA. The constructed wealth index values were then assigned to individuals based on accessible variables. The wealth index was divided into five groups: poorest (lowest poor 20%), poorer, middle, richer, and richest (top 20%). Furthermore, the wealth index recorded participants into three groups: 40% bottom (poor), middle 40% (middle) or top 20% (rich). Another control variable, the location of residence, was dichotomised as either urban or rural.

### Estimation strategies

#### Measuring and decomposing wealth-related inequalities

For the inequality analysis, utilisation of BCS services was performed across wealth quintiles. The standard measures of concentration index (CI) were employed to examine the magnitude of household wealth-related inequality and the trends in utilisation of BCS services across 14 developing countries. The CI was estimated as the covariance of the utilisation of BCS services and the proportional rank in wealth score distribution [47] as follows:

1 $$ CI=\frac{2}{n^2\overline{\mathrm{y}}\ }{\sum}_{i=1}^n{y}_i{r}_i $$

where CI is the concentration index, $\overline{y}$ is the mean utilisation of BCS services, r_i_ is the cumulative proportion that each individual represents over the total population once the latter has been ranked by the distribution of wealth score. The values of CI are bounded between $\overline{y}-1$ and $1-\overline{y}$; $\overline{y}-1\le \mathrm{CI}\le 1-\overline{y}$ when y is dichotomous [48, 49]. CI acquires a negative value when the curve lies above the line of equality, which indicates a disproportionately lower prevalence of BCS service utilisation among the poor (i.e., pro-poor). A positive value of CI signifies a higher concentration of health indicators among the rich (i.e., pro-rich). There is no socioeconomic inequality in the distribution of utilisation of BCS services (y) when the value of CI is zero and the concentration curve (CC) coincides with the 45° line. The dichotomous character of the utilisation of BCS services may result in unstable bounds in response to varying means; therefore, the normalised standard index was estimated to check the robustness of the estimation [50, 51]. In addition, when the outcome variable is dichotomous, the CI has to be corrected in order to allow comparisons between groups of individuals from different time periods that may show different levels of use of health services [52]. In the context of a dichotomous outcome variable, the Erreygers’s CI is the CI multiplied by four times the mean health or outcome of interest [53]. Erreygers’ suggested corrected CI can be expressed as:

2 $$ E=\frac{4\times \overline{y}}{y^{max}-{y}^{min}}\  CI $$

where *y*^*max*^ and *y*^*min*^ are the boundary of y (utilisation of BCS services). When the Erreygers’ corrected index is used, the decomposition of inequality is generally expressed as:

3 $$ E=4\times \sum \limits_k\left({\beta}_k^m{\overline{x}}_k\right){CI}_k+{GCI}_{\varepsilon } $$

This estimate produces an index that satisfies various attractive axiomatic properties for an inequality index, including the sign condition, scale invariance and mirror properties [53, 54]. The adjusted CI method allows for an examination of the causes of (and their corresponding contributions to) and levels of changes in inequalities in terms of the utilisation of BCS services [54]. In addition, multiple logistic regression was applied to measure the likelihood of utilisation of BCS services. Adjusted odds ratios (AORs) with a 95% confidence interval (CI) were estimated for identifying influencing factors on utilisation of BCS services at a 5% or lower level of significance. All statistical analyses were performed with Stata/SE-13 software (StataCorp, College Station, TX, USA).

## Results

### Distribution for the utilisation of BCS services by participant’s characteristics across geographical areas

Table 1 shows the distribution of the utilisation of BCS services. The population-weighted mean of the utilisation of BCS services across all countries was 15.41% (95% CI: 15.22–15.60%). There were wide variations in the percentage of the utilisation across geographical areas, for instance, 80.82% in Europe countries, 25.26% in South American countries, 16.95% in North American countries, 15.06% in Asia and 13.84% in African countries. A higher proportion of reproductive women (aged 40 to 44 years) utilised BCS services in Europe (73%), Africa (54%) and Asia (51%), compared with 43% in North America and 32% in South America. Female-headed households constituted 24.38% of the sample, approximately 51% or more of women had at least secondary education, and 61% of the women were screened in urban communities. About 41% of women lived in households with high SES status. Examining enabling factors, 55% of women had access to mass media communication. Also, 27% of households had health insurance, and 68% of women were overweight (Table 1).

**Table 1:** Distribution of the utilisation of breast cancer screening services

Participants characteristics	Africa	Asia	Europe	North America	South America	Overall
**Predisposing factors**	% (95% CI)	% (95% CI)	% (95% CI)	% (95% CI)	% (95% CI)	% (95% CI)
Age in years						
*40–44 years*	54.07 (52.82, 55.31)	50.93 (49.99, 51.86)	72.50 (68.10, 76.50)	43.27 (41.59, 44.96)	31.55 (28.37, 34.91)	50.39 (49.72, 51.05)
*≥ 45 years*	45.93 (44.69, 47.18)	49.07 (48.14, 50.01)	27.50 (23.50, 31.90)	56.73 (55.04, 58.41)	68.45 (65.09, 71.63)	49.61 (48.95, 50.28)
Educational level						
*No education*	8.28 (7.62, 8.99)	17.07 (16.38, 17.79)	0.20 (0.02, 1.63)	03.82 (03.22, 04.52)	02.33 (01.48, 03.67)	11.67 (11.25, 12.11)
*Primary*	41.21 (39.98, 42.44)	09.81 (09.27, 10.38)	46.72 (42.07, 51.44)	54.79 (53.09, 56.47)	26.40 (23.41, 29.62)	26.97 (26.39, 27.57)
*Secondary*	39.06 (37.85, 40.28)	47.88 (46.95, 48.82)	43.67 (39.07, 48.39)	29.96 (28.43, 31.54)	33.72 (30.48, 37.13)	42.03 (41.38, 42.69)
*Higher*	11.46 (10.69, 12.27)	25.24 (24.44, 26.06)	9.41 (7.00, 12.54)	11.43 (10.40, 12.56)	37.55 (34.20, 41.02)	19.32 (18.80, 19.85)
Head of the household						
*Female*	58.37 (57.14, 59.59)	87.69 (87.06, 88.29)	97.5 (95.52, 98.62)	62.46 (60.80, 64.09)	86.44 (83.84, 88.68)	75.62 (75.05, 76.19)
*Male*	41.63 (40.41, 42.86)	12.31 (11.71, 12.94)	2.50 (1.38, 4.48)	37.54 (35.91, 39.20)	13.56 (11.32, 16.16)	24.38 (23.81, 24.95)
Age of respondent at 1st birth						
*< 18 years*	21.75 (20.74, 22.8)	14.18 (13.52, 14.87)	0.41 (0.09, 1.79)	23.68 (22.26, 25.16)	14.37 (12.00, 17.11)	17.64 (17.13, 18.17)
*18–20 years*	34.87 (33.69, 36.07)	32.09 (31.20, 33.00)	13.67 (10.72, 17.27)	33.72 (32.13, 35.34)	26.03 (22.98, 29.34)	32.58 (31.95, 33.22)
*21–25 years*	31.3 (30.16, 32.47)	40.36 (39.42, 41.31)	59.82 (55.09, 64.37)	30.01 (28.47, 31.59)	31.41 (28.14, 34.87)	36.13 (35.49, 36.79)
*> 25 years*	12.07 (11.28, 12.91)	13.37 (12.72, 14.04)	26.10 (22.15, 30.48)	12.59 (11.51, 13.77)	28.19 (25.04, 31.57)	13.64 (13.18, 14.11)
Number of Births						
*< 4*	29.83 (28.7, 30.98)	19.68 (18.92, 20.46)	81.39 (77.44, 84.78)	42.77 (41.09, 44.46)	80.34 (77.39, 82.99)	29.82 (29.21, 30.44)
*4–5*	32.4 (31.24, 33.58)	35.53 (34.61, 36.46)	16.76 (13.53, 20.58)	32.72 (31.14, 34.33)	15.36 (12.99, 18.08)	33.03 (32.40, 33.67)
*> 5*	37.77 (36.57, 38.99)	44.80 (43.84, 45.76)	1.85 (0.93, 3.66)	24.52 (23.08, 26.01)	04.30 (03.08, 05.98)	37.15 (36.50, 37.80)
**Enabling factors**						
Mass media exposure						
*No*	23.29 (22.24, 24.36)	65.66 (64.77, 66.55)	49.03 (44.35, 53.74)	22.16 (20.78, 23.62)	15.76 (13.36, 18.50)	44.87 (44.21, 45.54)
*Yes*	76.71 (75.64, 77.76)	34.34 (33.45, 35.23)	50.97 (46.26, 55.65)	77.84 (76.38, 79.22)	84.24 (81.50, 86.64)	55.13 (54.46, 55.79)
Health Insurance coverage						
*No*	70.38 (69.23, 71.5)	82.87 (81.32, 84.31)	72.55 (68.15, 76.55)	70.03 (68.44, 71.56)	71.49 (69.23, 71.5)	72.82 (72.03, 73.60)
*Yes*	29.62 (28.50, 30.77)	17.13 (15.69, 18.68)	27.45 (23.45, 31.85)	29.97 (28.44, 31.56)	28.51 (28.50, 30.77)	27.18 (26.40, 27.97)
Nutritional status						
*Underweight*	4.33 (3.78, 4.96)	04.27 (03.84, 04.75)	1.12 (0.98, 2.23)	0.99 (0.70, 1.39)	4.23 (3.84, 4.75)	3.49 (3.22, 3.79)
*Normal weight*	35.23 (33.86, 36.63)	27.86 (26.87, 28.88)	36.28 (32.84, 41.94)	17.65 (16.38, 18.98)	27.86 (26.87, 28.88)	28.11 (27.42, 28.82)
*Overweight*	60.44 (59.01, 61.85)	67.86 (66.81, 68.90)	62.60 (58.07, 67.16)	81.37 (80.00, 82.66)	67.91 (66.81, 68.90)	68.39 (67.67, 69.11)
**Community**						
*Urban*	45.91 (44.67, 47.15)	65.54 (64.65, 66.42)	45.61 (40.97, 50.33)	71.95 (70.39, 73.45)	84.50 (81.78, 86.88)	61.20 (60.55, 61.85)
*Rural*	54.09 (52.85, 55.33)	34.46 (33.58, 35.35)	54.39 (49.67, 59.03)	28.05 (26.55, 29.61)	15.50 (13.12, 18.22)	38.80 (38.15, 39.45)
Economic status						
*Low*	27.68 (26.58, 28.8)	24.80 (24.01, 25.62)	32.82 (28.56, 37.39)	23.18 (21.78, 24.65)	25.69 (22.73, 28.89)	29.19 (28.59, 29.79)
*Moderate*	40.76 (39.54, 41.99)	33.59 (32.71, 34.48)	45.28 (40.65, 50.00)	38.11 (36.47, 39.78)	38.69 (35.32, 42.18)	30.01 (29.41, 30.63)
*High*	31.56 (30.42, 32.73)	41.61 (40.69, 42.53)	21.89 (18.24, 26.04)	38.71 (37.06, 40.38)	35.62 (32.32, 39.06)	40.80 (40.15, 41.46)
**Overall**	13.84 (13.53, 14.17)	15.06 (14.80, 15.32)	80.82 (77.27, 83.94)	16.95 (16.43, 17.48)	25.26 (23.76, 26.83)	15.41 (15.22, 15.60)

**Fig. 1:**
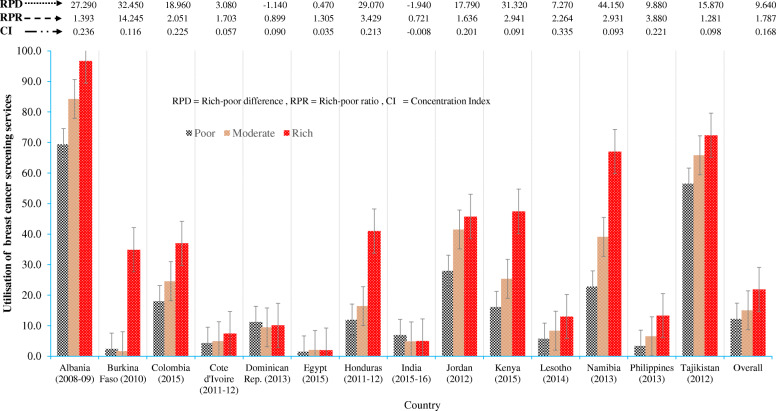
Unequal distribution of the use of breast cancer screening in 14-low resource countries

There were also wide variations in the percentage of the utilisation across countries, from over 81% in Albania to 10% or less in five countries: Cote d’Ivoire, Lesotho, India, Egypt and the Philippines (Additional file 1 Table A1). The utilisation of BCS services was unequally distributed according to wealth (Fig. 1). The highest utilisation of the BCS was found in the highest wealth quintile in Albania (95%), Tajikistan (72%), Namibia (67%), and Kenya (47%). However, a high degree of inequality in utilising BCS services was observed when using the rich-poor ratio (RPR), the rich-poor difference (RPD) and the concentration indices (CI). The RPR was highest in Burkina Faso (14.5), Philippines (3.9), Honduras (3.4) and Kenya (2.9), with the lowest values in India (0.7), and the Dominican Republic (0.9). The concentration indices were highest in Lesotho (CI = 0.335), Albania (CI = 0.236), The Philippines (CI = 0.221) and Honduras (CI = 0.213). Moreover, these results signified that women in higher SES households utilised more BCS services than women living in disadvantaged households. This scenario was similar in the majority of countries.

### Factors influencing utilisation of BCS screening services

Several factors influence the utilisation of BCS services (Table 2). For example, age (OR = 0.97), year of schooling (OR = 1.03), living in rural locations (OR = 0.59) and higher wealth (OR = 1.07) had a significant impact on accessing and consuming BCS services. Other factors significantly driving higher rates of utilisation of BCS services, including being female-headed households (OR = 1.06), being a woman in the richest households (OR = 1.07) and having access to mass media communications (OR = 1.59).

**Table 2:** Inequality decomposition of the Erreygers’s concentration index for utilising of breast cancer screening services

Variables	Odds ratio (OR) (95% CI)	Elasticity	Erreygers’s concentration index (CI)	Relative contribution to the Erreygers’s CI, (%)
**African countries**				
Age (year)	1.00 (0.98, 1.01)	− 0.21	0.20	6.33
Education (year)	0.95 (0.93, 0.96)	0.27	− 0.01	12.16
Age at 1st birth (year)	0.97 (0.96, 0.98)	− 0.68	0.85	5.09
Number of births (n)	0.98 (0.96, 1.00)	− 0.10	− 0.25	− 0.86
Sex of household head (ref = male)	1.76 (1.63, 1.90)	0.73	− 0.26	5.63
Mass media communication (ref = no)	2.34 (2.16, 2.54)	0.43	0.34	6.15
Health insurance coverage (ref = no)	2.03 (1.86, 2.20)	0.09	0.24	−0.17
Body mass index	0.96 (0.95, 0.96)	−0.26	0.62	14.53
Community (ref = rural)	0.91 (0.83, 0.99)	0.15	−0.68	8.83
Wealth score	1.19 (1.15, 1.23)	0.50	0.67	23.34
Total				81.04
**Asian countries**				
Age (year)	0.93 (0.91, 0.96)	−0.26	0.75	10.53
Education (year)	1.18 (1.12, 1.23)	0.63	0.26	18.33
Age at 1st birth (year)	1.03 (1.01, 1.05)	0.61	−0.09	−0.42
Number of births (n)	1.20 (1.15, 1.26)	0.90	−0.88	6.52
Sex of household head (ref = male)	1.51 (1.25, 1.83)	0.47	−0.02	9.06
Mass media communication (ref = no)	1.08 (0.87, 1.35)	0.02	0.19	0.03
Health insurance coverage (ref = no)	0.53 (0.43, 0.64)	−0.15	−0.05	0.01
Body mass index	1.02 (1.01, 1.03)	0.47	0.69	10.38
Community (ref = rural)	0.73 (0.62, 0.86)	−0.51	−0.31	6.26
Wealth score	0.98 (0.91, 1.05)	−0.06	0.87	25.27
Total				85.95
**European countries**				
Age (year)	1.03 (0.93, 1.13)	0.29	0.83	7.25
Education (year)	0.90 (0.80, 1.02)	−0.58	−0.92	14.85
Age at 1st birth (year)	1.00 (0.92, 1.09)	0.01	0.68	−6.06
Number of births (n)	0.88 (0.71, 1.09)	−0.37	− 0.20	16.57
Sex of household head (ref = male)	0.80 (0.16, 4.10)	− 0.23	0.02	−4.04
Mass media communication (ref = no)	0.55 (0.34, 0.90)	− 0.29	0.21	− 0.49
Health insurance coverage (ref = no)	1.94 (0.96, 3.93)	0.20	0.34	11.54
Body mass index	1.01 (0.98, 1.04)	0.28	0.31	5.69
Community (ref = rural)	1.18 (0.57, 2.47)	0.25	−0.90	−1.81
Wealth score	1.58 (1.19, 2.09)	0.45	0.43	37.40
Total				80.92
**North American countries**				
Age (year)	1.08 (1.07, 1.10)	0.93	0.40	17.86
Education (year)	1.04 (1.02, 1.06)	0.20	−0.03	13.05
Age at 1st birth (year)	1.01 (1.00, 1.02)	0.34	0.21	−6.80
Number of births (n)	0.95 (0.93, 0.98)	−0.44	−0.15	12.07
Sex of household head (ref = male)	1.01 (0.93, 1.10)	0.12	0.02	3.02
Mass media communication (ref = no)	1.00 (0.91, 1.10)	−0.08	0.46	−0.30
Health insurance coverage (ref = no)	0.80 (0.73, 0.88)	0.01	0.16	14.01
Body mass index	1.01 (1.01, 1.02)	−0.06	0.26	−6.65
Community (ref = rural)	0.90 (0.81, 0.99)	0.00	−0.30	4.69
Wealth score	1.35 (1.3, 1.41)	0.03	0.65	35.83
Total				86.77
**South American countries**				
Age (year)	1.27 (1.23, 1.31)	0.53	0.12	16.46
Education (year)	0.98 (0.93, 1.03)	−0.09	0.39	−0.28
Age at 1st birth (year)	0.99 (0.97, 1.01)	−0.12	2.19	−2.10
Number of births (n)	0.93 (0.86, 1.00)	−0.25	−1.53	13.03
Sex of household head (ref = male)	1.01 (0.77, 1.34)	0.02	−0.01	6.00
Mass media communication (ref = no)	0.92 (0.72, 1.18)	−0.06	0.18	−0.09
Health insurance coverage (ref = no)	0.81 (0.73, 0.88)	0.01	0.16	16.01
Body mass index	1.03 (1.01, 1.06)	−0.06	0.26	−2.65
Community (ref = rural)	1.31 (0.95, 1.81)	0.36	−0.62	−1.81
Wealth score	1.36 (1.24, 1.49)	0.75	0.73	36.28
Total				80.85
**Overall**				
Age (year)	0.97 (0.96, 0.98)	−0.27	0.36	13.34
Education (year)	1.03 (1.02, 1.04)	0.29	0.33	0.67
Age at 1st birth (year)	1.08 (1.08, 1.09)	1.02	0.32	5.59
Number of birth (n)	1.14 (1.13, 1.15)	0.40	−0.71	−5.50
Sex of household head (ref = male)	1.06 (1.02, 1.11)	0.58	−0.03	− 0.13
Mass media communication (ref = no)	1.59 (1.53, 1.66)	0.14	0.13	0.15
Health insurance coverage (ref = no)	0.99 (0.93, 1.07)	0.60	0.01	15.00
Body mass index	1.02 (1.01, 1.02)	0.44	0.64	14.10
Community (ref = rural)	0.59 (0.56, 0.61)	−0.32	−0.46	1.19
Wealth score	1.07 (1.05, 1.09)	0.21	0.10	35.12
Total				79.53

### Decomposition of the utilisation of BCS services inequalities

Table 2 present the results from the decomposition analysis of the utilisation of BCS services inequalities which indicate the effects and contributions of various socioeconomic and demographic factors. The table presents the results of elasticity analysis, the concentration index (CI) of the regressors, and the percentage contribution of regressors to the inequality of utilisation of BCS services. Higher elasticity values resulted for years of schooling, female-headed household, number of childbirths, access to mass media exposure, and wealth score determinants of utilisation of BCS services. The higher values of elasticity signified that these factors have a significant impact on the utilisation of BCS services. Overall, this study also found that women’s age (13.34%), health insurance coverage (15%), body mass index (14.10%) and wealth score (35%) made a significant contribution to BCS services utilisation inequality explaining a higher proportion of the total inequality in utilisation of BCS services. This scenario was very similar across geographical areas, for instance: in African countries, women’s age (6.33%), years of schooling (12%), access to mass media communication (6%), body mass index (15%), urban communities (9%), and wealth scores (23.34%) were important contributors regarding inequality of BCS services. Among the variables, the greatest contributions towards inequality were observed in the utilisation of BCS services in the context of Asian, Europe, North America and South America’s countries.

## Discussion

This study investigated the extent of socio-economic inequality in the utilisation of BCS services among reproductive women by estimating their utilisation rates. It quantified each contribution to the inequality gap via predisposing, enabling, and community factors. This enabled decomposition of the utilisation of BCS services. The regression-based decomposition technique enabled the contribution of the gap characteristics to potential factors to be estimated as the proportion attributable to examine the inequality of the utilisation of BCS services. The main findings were that a high degree of socio-economic inequality in the utilisation of BCS services exists in these 14 developing countries across dispersed geographical areas. Women with high SES were more likely to access more BCS services than their low SES counterparts. This gap in the utilisation of BCS among participants with high and low SES was statistically significant for all countries. In general, the findings indicated substantial heterogeneity, both in the magnitude of inequality and the contributions of different factors to inequality. A further striking finding is that with countries in the same geographic settings and a similar level of economic development had significantly different outcomes. The most significant decomposing factors of inequality in the utilisation of BCS services were women’s age, health insurance coverage, body mass index and wealth score. These factors made a significant contribution to BCS services, resulting in a higher proportion of the total inequality in utilisation of BCS services.

Overall, the decomposition analysis showed that the participation rates in utilisation of the BCS services were concentrated in women with the high wealth scores. Significantly, those with high wealth scores utilised BCS services 1.40 times as much as those in low SES groups. This signifies that participants in richer households were more likely to utilise the BCS services than their poorer counterparts. This association has also been reported previously in other studies in different nations [12, 13, 55–57]. These studies had identified that socioeconomic status was the main driver contributing to the unequal distribution of participation in the BCS services. Women from high SES households access more BCS services due to higher education levels. In addition, participants from high SES households were poorly correlated with negative perceptions about BCS services. Some previous studies have shown that ease of access to BCS services was likely to facilitate use by low SES participants [20, 21, 58]. Service provision led to a reduction in plausible barriers to participation in BCS services even for that working low-paying, menial jobs; having a limited time or opportunity to participate in screening programs; or with a lack of access to relevant information [20, 21, 59]. The consistency of this finding suggests that exploring or expanding public BCS programs especially in low-resource countries will be beneficial in increasing the participation rate of women from lower SES groups seeking BCS services.

The results also found that a lack of mass media exposure in households reduced participation in BCS services. In general, participants in low SES households had limited access to mass media communication compared with high SES households [59]. Media communications were commonly cited as the primary vehicle for improving cancer screening-related awareness because they broadcast relevant health messages and promote behavioural health change [60–63]. Even though the majority of the studied countries have effective mass media environments, some initiatives should be undertaken to extend this beyond existing national and regional level programs. Routine health education sessions, stakeholders engagement, community demonstrations (e.g., role-play and skits), and courtyard meetings with household heads and women should be undertaken.

The results also showed that household location, i.e. rural vs. urban, influences inequality in accessing BCS services. Participants living in urban settings, and women living in the middle SES households were more likely to utilise BCS services compared women living in middle SES households in rural settings. Other studies have also found that participants in urban settings were more likely to utilise BCS services compared with their rural counterparts [13, 20, 23, 56, 57, 59]. Several factors may contribute to this inequality, for instance, women from rural communities often have limited access to resources and health facilities [59]; frequently there is poor health service delivery in rural communities [58, 59]; there is a lack of community awareness [10], and there are lower living standards and poor access to health services [55, 59, 60]. The result confirms that uninsured participants have significantly lower usage rates of BCS services compared with insured participants. This finding has shown consistency with other prior studies which found that participants in uninsured households were less likely to access BCS services than those in insured households [60–69]. Lack of health insurance programs for rural women is a significant impediment in many low-resource countries hence, the cost and access of the BCS services adversely affect the poorest [59, 60]. A screening intervention can be successful only for services which are available, affordable, and acceptable to the individual, the community, and the jurisdiction of interest. Since women in insured households can afford more health care services via their health insurance [59–64]. These findings suggest that health care financing mechanisms could be introduced especially in low-resource settings, to reduce the high levels of inequality evidenced and to increase BCS service access. In addition, media communications are commonly cited as the primary vehicle for improving cancer screening-related awareness because they broadcast relevant health messages and promote behavioural health change [66–68]. Even though the majority of the studied countries have effective mass media environments, some initiatives should be undertaken to extend this beyond existing national and regional level programs. Routine health education sessions, stakeholders engagement, campaigns, demonstrations (e.g., posters or leaflets), and courtyard meetings with household heads and women should be undertaken.

This study has some limitations. The study design was cross-sectional, and thus, the causal inference was limited due to a lack of information and study design. In the DHS survey, some potential factors related to BCS information were not captured, which may affect the estimates obtained. Further research needs to examine the causal inference for utilising BCS as well as identify potential barriers and challenges that prevent socio-economically disadvantaged women from accessing BCS services. All findings were generated based on individual self-reported data, which is an issue in terms of recall and social desirability bias. Future studies might confirm these results. The ‘poor wealth’ quintile was compared with the ‘rich wealth’ quintile under the premise that the poor would experience an increase in the uptake of screening to the levels of rich. Although there might be a problem of over-screening amongst the rich, this premise would still be valid because a survey question asking an individuals’ participation in screening services was used. The participants’ dichotomous responses to the use of BCS services did not follow descriptive responses to allow for cross-validation of qualitative data. In the analytical exploration, the authors have attempted to correct for bias from missing data by using a multiple imputation method. However, this technique may not adequately address all the bias in the DHS data.

Countries asked a wide range of questions to capture information about the disease and screening knowledge, practices, or outcomes. The most frequently assessed topics were whether the respondent had been screened for breast cancer and the timing or frequency of this screening. To guide national efforts to reduce the effects of cancers, surveys need to provide specific and measurable information about both the quantity and the quality of cancer screenings. However, questions about follow-up and treatment of screened women were rare and missing a component of the screening process that is necessary to achieve reductions in cancer incidence and mortality. Very few surveys have incorporated these essential questions, which will enable countries to evaluate the effectiveness of their cancer screening practices beyond estimates of the proportion of eligible women screened.

Despite these limitations, the DHS provides the first opportunity to investigate the distribution of BCS utilisation and the magnitude of wealth-related inequalities across a large set of low-resource countries with a comparison of geographical areas. The findings of this study provide a solid foundation for further research while highlighting the need to improve the quality of services and the frequency of monitoring of breast cancer screening and control efforts worldwide.

## Conclusions

This study investigated the distribution and utilisation of the BCS services in 14 low-resource countries and examined the magnitude of the gap in inequality in utilising BCS services. It also investigated the factors that contribute most to this identified gap. The utilisation of BCS services was comparatively very low in low-resource countries along with a high degree of inequality evidenced by explaining the most significant decomposing factors. Exploring or expanding public BCS programs especially in low-resource countries will be extremely beneficial in increasing the participation rate of women from lower SES groups. Improving the effective coverage of BCS services or introducing alternative effective ways to decrease breast cancer mortality and incidence rates worldwide would have a considerable impact on decreasing the disease’s burden as well as overall health inequalities. A single strategy will not work everywhere, making it important to consider multiple strategies across- and likely within countries might be effective. Finally, these findings can assist policymakers to develop risk-pooling financing mechanisms that might ensure to reduce out-of-pocket payment for the utilisation of the BCS services, access to service available, affordable, and acceptable to the individual and the jurisdiction of interest. These might contribute to a decrease in the high levels of inequality evidenced in accessing the BCS services.

## Supplementary information

**Additional file 1: Table A1.** Country-specific utilisation of breast cancer screening services (*N* = 140,974).

## Data Availability

The DHS data are publicly accessible and were made available to us upon request by Measure DHS (https://dhsprogram.com/data/available-datasets.cfm).

## Abbreviations

BCS: Breast Cancer Screening
RPR: Rich-poor ratio
RPD: Rich-poor difference
CI: Concentration indices (CI)
PCA: Principal components analysis
DHS: Demographic health survey
SES: Socioeconomic status
