# Supplementary material for: Wealth stratified inequalities in service utilisation of breast cancer screening across the geographical regions: a pooled decomposition analysis
Source: Arch Public Health. 2020 Jun 10;78:32. doi: 10.1186/s13690-020-00410-5 (PMC7285540; doi:10.1186/s13690-020-00410-5)
Supplement: Supplementary file 1 — Additional file 1: Table A1. Country-specific utilisation of breast cancer screening services (N = 140,974). [file 13690_2020_410_MOESM1_ESM.docx]

Additional file 1 Table A1. Country-specific utilisation of breast cancer screening services (N = 140,974)

| **Participants characteristics** | Albania (2008-09) | Burkina Faso (2010) | Colombia (2015) | Cote d'Ivoire (2011-12) | Dominican Rep. (2013) | Egypt (2015) | Honduras (2011-12) | India  (2015-16) | Jordan (2012) | Kenya (2015) | Lesotho (2014) | Namibia (2013) | Philippines (2013) | Tajikistan (2012) |
| --- | --- | --- | --- | --- | --- | --- | --- | --- | --- | --- | --- | --- | --- | --- |
| **Predisposing factors** |  |  |  |  |  |  |  |  |  |  |  |  |  |  |
| Age in years |  |  |  |  |  |  |  |  |  |  |  |  |  |  |
| *40-44 years* | 79.99 | 11.49 | 15.85 | 4.69 | 9.92 | 2.44 | 16.91 | 6.34 | 35.85 | 25.38 | 9.66 | 37.39 | 6.10 | 64.51 |
| *≥ 45 years* | 83.09 | 8.84 | 34.77 | 6.04 | 10.63 | 1.22 | 23.93 | 5.10 | 37.72 | 23.75 | 7.31 | 38.24 | 7.31 | 62.78 |
| Educational level |  |  |  |  |  |  |  |  |  |  |  |  |  |  |
| *No education* | 27.93 | 4.63 | 21.93 | 4.70 | 1.51 | 1.05 | 11.27 | 6.36 | 25.49 | 6.83 | 3.87 | 19.51 | 2.98 | 24.74 |
| *Primary* | 72.92 | 20.10 | 21.31 | 5.32 | 11.38 | 1.24 | 16.16 | 3.95 | 27.58 | 22.41 | 6.97 | 28.40 | 4.00 | 58.29 |
| *Secondary* | 88.42 | 42.96 | 21.16 | 5.70 | 11.22 | 2.32 | 32.32 | 4.77 | 35.99 | 36.52 | 10.32 | 47.84 | 6.57 | 59.82 |
| *Higher* | 98.39 | 80.69 | 36.82 | 32.20 | 8.28 | 4.02 | 47.45 | 7.08 | 45.19 | 59.82 | 18.28 | 63.48 | 12.51 | 83.39 |
| Head of the household |  |  |  |  |  |  |  |  |  |  |  |  |  |  |
| *Male* | 80.71 | 10.53 | 24.91 | 5.54 | 9.21 | 1.96 | 20.03 | 5.22 | 37.35 | 26.00 | 8.44 | 38.56 | 6.86 | 63.70 |
| *Female* | 85.38 | 13.32 | 27.78 | 4.09 | 11.37 | 0.80 | 21.06 | 8.01 | 28.87 | 22.83 | 8.54 | 37.17 | 7.83 | 63.72 |
| Age of respondent at 1st birth |  |  |  |  |  |  |  |  |  |  |  |  |  |  |
| *<18 years* | 58.90 | 6.98 | 22.72 | 3.67 | 6.99 | 1.81 | 16.25 | 4.85 | 32.62 | 19.93 | 5.90 | 34.88 |  | 54.34 |
| *18-20 years* | 78.62 | 9.74 | 21.62 | 4.27 | 8.39 | 1.12 | 19.63 | 5.72 | 35.15 | 25.00 | 8.37 | 35.92 | na | 63.62 |
| *21-25 years* | 80.36 | 8.16 | 24.35 | 5.29 | 14.47 | 2.00 | 23.26 | 5.93 | 39.32 | 27.88 | 8.16 | 39.78 |  | 64.03 |
| *>25 years* | 82.75 | 25.71 | 30.11 | 19.91 | 13.27 | 2.84 | 31.79 | 8.36 | 36.09 | 34.35 | 18.64 | 42.04 |  | 65.20 |
| Number of Births |  |  |  |  |  |  |  |  |  |  |  |  |  |  |
| *<4* | 82.93 | 25.01 | 26.98 | 10.03 | 11.66 | 1.82 | 31.46 | 4.42 | 40.32 | 45.63 | 11.03 | 50.19 | na | 67.08 |
| *4-5* | 74.25 | 21.26 | 21.57 | 4.36 | 10.03 | 1.68 | 20.06 | 6.28 | 41.15 | 32.22 | 6.26 | 37.70 |  | 66.19 |
| *>5* | 61.36 | 3.83 | 16.02 | 4.85 | 7.66 | 2.11 | 13.44 | 7.87 | 33.86 | 16.65 | 8.67 | 29.31 |  | 57.21 |
| **Enabling factors** |  |  |  |  |  |  |  |  |  |  |  |  |  |  |
| Mass media exposure |  |  |  |  |  |  |  |  |  |  |  |  |  |  |
| *No* | 82.21 | 4.49 | 21.06 | 2.59 | 10.35 | 1.61 | 17.36 | 5.60 | 33.92 | 16.04 | 7.61 | 25.17 | 5.78 | 63.27 |
| *Yes* | 79.54 | 12.37 | 26.24 | 7.26 | 10.51 | 2.43 | 21.08 | 5.67 | 41.52 | 28.85 | 9.08 | 42.63 | 7.87 | 64.99 |
| Health Insurance coverage |  |  |  |  |  |  |  |  |  |  |  |  |  |  |
| *No* | 77.38 | 10.31 | na | 5.14 | 10.73 | 1.52 | 18.05 | 6.58 | na | 20.63 | 7.81 | 31.56 | na |  |
| *Yes* | 91.62 | 27.65 |  | 8.17 | 10.10 | 4.17 | 41.61 | 3.30 |  | 43.49 | 34.01 | 60.22 |  |  |
| Nutritional status |  |  |  |  |  |  |  |  |  |  |  |  |  |  |
| *Underweight* | 0.00 | 8.99 | na | 0.81 | 16.63 | 0.00 | 9.53 | 5.71 | 40.13 | 16.39 | 0.00 | 15.93 | na | 59.08 |
| *Normal weight* | 78.13 | 3.85 |  | 1.96 | 8.06 | 0.97 | 15.50 | 5.89 | 35.10 | 20.33 | 7.32 | 32.42 |  | 65.30 |
| *Overweight* | 83.50 | 27.11 |  | 5.86 | 10.81 | 1.92 | 21.80 | 5.40 | 35.87 | 31.01 | 9.38 | 48.50 |  | 62.92 |
| **Community** |  |  |  |  |  |  |  |  |  |  |  |  |  |  |
| *Urban* | 89.51 | 27.11 | 27.21 | 7.17 | 10.68 | 1.71 | 26.18 | 5.64 | 37.75 | 36.32 | 10.83 | 51.38 | 8.83 | 69.98 |
| *Rural* | 74.74 | 3.77 | 18.17 | 3.84 | 9.24 | 1.92 | 13.13 | 5.62 | 31.52 | 21.06 | 7.68 | 26.49 | 5.49 | 61.13 |
| Economic status |  |  |  |  |  |  |  |  |  |  |  |  |  |  |
| *Low* | 69.38 | 2.45 | 18.04 | 4.38 | 11.28 | 1.54 | 11.97 | 6.96 | 27.98 | 16.14 | 5.75 | 22.86 | 3.43 | 56.48 |
| *Moderate* | 84.22 | 1.71 | 24.61 | 5.00 | 9.49 | 2.11 | 16.46 | 4.91 | 41.54 | 25.38 | 8.40 | 39.15 | 6.59 | 65.79 |
| *High* | 96.67 | 34.90 | 37.00 | 7.46 | 10.14 | 2.01 | 41.04 | 5.02 | 45.77 | 47.46 | 13.02 | 67.01 | 13.31 | 72.35 |
| **Overall** | 80.82 | 10.56 | 25.26 | 5.27 | 10.28 | 1.84 | 20.36 | 5.63 | 36.67 | 24.61 | 8.47 | 37.80 | 7.08 | 63.70 |
| **Observations** | 539 | 473 | 3,075 | 5,382 | 6,643 | 16,973 | 12,975 | 43,502 | 18,255 | 11,847 | 3,993 | 6,065 | 9,384 | 1,866 |

Note: na = not available, all estimates were sample weight adjusted
